# DiI tracing of the hypothalamic projection systems during perinatal development

**DOI:** 10.3389/fnana.2014.00144

**Published:** 2014-12-04

**Authors:** Irina G. Makarenko

**Affiliations:** Laboratory of Cellular and Molecular Basis of Histogenesis, Koltzov Institute of Developmental Biology, Russian Academy of SciencesMoscow, Russia

**Keywords:** DiI, hypothalamus, intrahypothalamic, septal, mammillary, mammillothalamic, mammillotegmental, rat

## Abstract

The hypothalamus is the higher neuroendocrine center of the brain and therefore possesses numerous intrinsic axonal connections and is connected by afferent and efferent fiber systems with other brain structures. These projection systems have been described in detail in the adult but data on their early development is sparse. Here I review studies of the time schedule and features of the development of the major hypothalamic axonal systems. In general, anterograde tracing experiments have been used to analyze short distance projections from the arcuate and anteroventral periventricular nuclei (Pe), while hypothalamic projections to the posterior and intermediate pituitary lobes (IL) and median eminence, mammillary body tracts and reciprocal septohypothalamic connections have been described with retrograde tracing. The available data demonstrate that hypothalamic connections develop with a high degree of spatial and temporal specificity, innervating each target with a unique developmental schedule which in many cases can be correlated with the functional maturity of the projection system.

## Introduction

The hypothalamus is an important structure of the brain positioned as a higher part of the vegetative nervous system and a part of the limbic system. It is involved in realization of numerous neuroendocrine, endocrine, somatomotor and behavioral functions which help an organism to survive and adopt to the environment (Swanson, 2000; Simerly, 2004). The background of such complex functioning lies in multiple intrinsic and external axonal connections. The adult hypothalamic projection systems have been examined in modern times by means of axonal tracing techniques based on stereotaxic injections (Vercelli et al., 2000; Makarenko, 2008) which cannot be applied to the study of embryos or early postnatal animals. For this reason, the early formation of hypothalamic connections is unsufficiently known. However, the beginning of axogenesis and tract formation are important events, as they begin the complex task of forming the network on which neural function depends (Easter et al., 1993). The formation of axonal connections and the time schedule of these processes in different neural systems have been considered to be the least well-known events in brain development (Keshavan and Murray, 1997) but such knowledge may be important to investigate potential therapeutic interventions, for instance in pathological obesity (Grayson et al., 2006). As the hypothalamus is an integrator of homeostatic processes required for survival, disruption of its development may cause pathological conditions in the adult (Caqueret et al., 2005). Here I will review the current knowledge concerning the time schedule and specific features of formation of several hypothalamic axonal systems using the most commonly used available method, DiI tracing.

## Methodological considerations

Carbocianine dye tracing was introduced for studies of the brain connections since first works of Honig and Hume (1986, 1989) and Godement et al. (1987). Although there are several carbocyanine dyes (whose advantages and disadvantages I have compared in a previous review (Makarenko, 2008)) one of them has been used predominantly for tracing developing hypothalamic connections: 1,1′-dioctadecyl-3,3,3′3,3′ -tetramethyl-indocarbocyanine perchlorate (DiI) (Molecular Probes, Eugene, OR). It diffuses along the lipid layer of the axonal membrane both in the anterograde and retrograde directions. The general steps of DiI tracing have been described before (see for instance (Molnar et al., 2006; Makarenko, 2008)). Anterograde DiI tracing can only be used in hypothalamic regions which do not have reciprocal connections as is the case for the arcuate nucleus projections to the paraventricular hypothalamic nucleus (Bouret et al., 2004). The existence of reciprocal connections makes the interpretation of the results difficult as it is not possible to find out if fibers were labeled anterogradely or retrogradely. Unwanted retrograde labeling of axons ending on our nucleus of interest can be assumed when neuronal cell bodies are found labeled in a different nucleus. Sometimes, authors deal with this fact by indicating that retrogradely labeled neurons have been omitted for clarity (Hutton et al., 1998) or are not numerous (Polston and Simerly, 2006). To make things worse, neuronal cell bodies are only poorly labeled at 37°C (the temperature at which DiI-labeled brains are stored according to many current protocols), making retrogradely labeled axons difficult to recognize. In my experience however, storage at room temperature usually labels numerous cell bodies with full dendritic trees clearly recognizable (Makarenko, 2008). For these reasons, the only sure method to evaluate anterogradely labeled fibers ending on a specific nucleus is to visualize labeled terminal arborizations and synaptic boutons.

The small dimensions of the hypothalamic nuclei at early developmental stages require very precise application of the marker, sometimes even single crystals of appropriate size were used (Hutton et al., 1998; Bouret et al., 2004). The need for long storage of the brain with DiI application at room temperature is one of the restrictions of this kind of tracing axonal connections but it provides excellent results. We have used storage times of at least 3–4 months at room temperature for the fetal rat brains and up to 6 months for rat postnatal material. The retrograde DiI labeling was very well preserved even after 1–2 years storage in 4% paraformaldehyde (Makarenko, 2007). Storage of the brain with DiI insertion at +37–40°C accelerates labeling (Lukas et al., 1998) and can give excellent results (Bouret et al., 2004; Polston and Simerly, 2006). In our hands however, these temperatures have failed to give satisfactory results. Sometimes it takes 12 or 26 weeks even with the heating (Bader et al., 2012).

Sectioning and coverslipping are also important. Thick vibratome sections (80–100 μm) sections are the most appropriate for the analysis of DiI tracing results using conventional or confocal fluorescence microscopy. Frozen sections can not be recommended for the DiI tracing because of the fast lateral diffusion of the marker from the axon in any type of the mounting medium. This can be overcome only by mounting and drying cryocut sections without coverslipping (Makarenko et al., 2001). As for the medium for mounting the sections, it is common to use buffered glycerol (Magoul et al., 1994; Hutton et al., 1998; Bouret et al., 2004), but others consider Mowiol (Calbiochem, Germany) more convenient (Makarenko, 2007; Alpeeva and Makarenko, 2009; Bader et al., 2012). Sections coverslipped using Mowiol can be stored for a long time without bleaching of the label and drying of the medium (Makarenko, 2008).

Additional control DiI insertions in the regions adjacent to the studied source of projections are necessary and useful because they usually resulted in labeling patterns that are distinct from those in the main experiments. They help in the analysis of specificity and interpretation of the results (Bouret et al., 2004; Alpeeva and Makarenko, 2007). The importance of methodological details and careful analysis in DiI tracing studies can be demonstrated on one example. Kouki and Yamanouchi (2007) set out to analyze the postnatal development of the lateral septal projections to the midbrain, and found septo-hypothalamic connections developing on 8 week-old rats. However, several poor methodological decisions make it difficult to evaluate their results. By using a DiI solution in dimethylformamide on the lateral septum, they labeled a wide region including not only the lateral septal nucleus but also adjacent regions; they incubated the tissue under excessive temperature (+40°C), and their analysis was performed on sections that were too thick (200 μm) and photographed at too low magnification with conventional fluorescence microscopy. As a result, they described only few fibers from the lateral septum in the preoptic region at birth and did not find labeled neurons there. Conversely, our own data obtained after similar experiments demonstrates that the first neurons of the lateral septal nucleus send axons to the preoptic area of the hypothalamus as early as E14–E15 and on E18–E20 these neurons have prominent ramified dendrites (Makarenko, 2007). According our preliminary results reversed hypothalamic projections to lateral septum also developed prenatally at least from E18 on (Makarenko, 2008). Perhaps the poor quality of the labeling in the mentioned work was associated with age of rats. Based on our work on the innervation of the anterior thalamic nuclei (Alpeeva and Makarenko, 2009), we support the view that DiI tracing works better on the prenatal and early postnatal material than in adults (Balthazart et al., 1994; Vercelli et al., 2000). The quality of the preterminal labeling in the thalamus decreased significantly after postnatal day (P)20 and on P60 distinct terminal arborizations could not be distinguished at all.

## Anterograde tracing of developing hypothalamic connections

The first DiI tracing studies of the hypothalamic connections were performed on adult rats. It was shown that projections from the arcuate nucleus to the bed nucleus of the stria terminalis follow through the stria terminalis and this route coincides with the orientation of ACTH-immunoreactive axons (Magoul et al., 1994). DiI was used also in studies analyzing the integration in the host brain of fetal septal-preoptic grafts (Silverman et al., 1992). Retrograde DiI labeling has also been used in combination with immunocytochemical visualization of gonadotropin-releasing hormone (GnRH) in the hypothalamic neurons projecting to the mediobasal hypothalamus in postnatal Djungarian hamsters (Buchanan and Yellon, 1993). The authors noted the difficulties of such combination because the saponin treatment necessary for their immunocytochemical staining diminished DiI labeling. They described a decreasing number of GnRH neurons projecting to the median eminence in the process of sexual maturation of Djungarian hamster. Such a result may be due to the fact that visualization of DiI-labeled neurons in the adult brain is worse than in the early stages of development (Balthazart et al., 1994; Vercelli et al., 2000; Makarenko, 2008). A special modification of the method has been proposed for adult human material (Sparks et al., 2000).

### Suprachiasmatic nucleus

Anterograde DiI tracing of the suprachiasmatic nucleus connections in the hamster has revealed very rapid and simultaneous development of the efferents to the principal targets known to be innervated by the adult suprachiasmatic nucleus (medial preoptic nucleus, the paraventricular nucleus (PV) of the thalamus, the parvicellular division of the hypothalamic PV, the subparaventricular zone, the dorsomedial nucleus of the hypothalamus, the ventral lateral septum, the intergeniculate leaflet, the bed nucleus of the stria terminalis) on the first postnatal day (P1) with significant increase on P2–P3 (Müller and Torrealba, 1998). These projections begin to form before the ontogenetic neuron death on P3. Retinal afferents reach the suprachiasmatic nucleus later only on P6–P7.

### Bed nucleus of the stria terminalis

Projections from the bed nucleus of the stria terminalis to the anteroventral periventricular nucleus of the preoptic region are established between postnatal day 9 (P9) and P10 in male rats but not in females although projections from the bed nucleus of stria terminalis (BSTp) to the preoptic region were revealed earlier on P4–P7 both in males and females (Hutton et al., 1998).

### Arcuate nucleus of the hypothalamus

Special attention was paid to the hypothalamic circuitry that modulates feeding and energy expenditure as the disturbances in their normal formation during the critical periods of the perinatal development may have long-term consequences on feeding behavior and body weight management (Grove et al., 2005). As the arcuate nucleus is one of the most important hypothalamic nuclei involved in regulation of body weight and responding to leptin signals in adults but not during two postnatal weeks, specific attention was paid for its connections with other hypothalamic regions. The study of their development has been performed on postnatal mice using anterograde DiI tracing (Bouret et al., 2004) because early postnatal administration of leptin did not cause inhibition of food intake (Bouret, 2010). The authors found the anatomical basis for this fact: arcuate nucleus projections to the dorsomedial and paraventricular hypothalamic nuclei and lateral hypothalamic area are immature at birth and develop from P6, P8 and P10 on, reaching an adult-like pattern at P16–P18. Bouret et al. (2004) attempted to quantify the innervation of these hypothalamic nuclei using confocal images. Such experiments are difficult, since it is not easy to standardize marker application on the brains at different developmental stages. An additional problem is that DiI labels all terminal arborizations of the axons but not only nerve terminals. Regardless of this their results provide a very good demonstration of increasing fiber density in the target nuclei. Additionally they found that arcuate nucleus axons reach the lateral hypothalamic area on P10, the medial preoptic nucleus on P12 and later they reach more rostral extrahypothalamic regions (the BSTp, paraventricular nucleus of the thalamus, or ventral part of the lateral septum). Thus all arcuate nucleus projections are formed primarily during the second postnatal week and appeared to be fully developed by P18. These results explain why there were no labeled neurons in the arcuate nucleus following DiI insertion in the lateral septal nucleus of the rat fetuses (Makarenko, 2007).

### Anteroventral periventricular nucleus of the hypothalamus

Later a new methodological approach for quantifying of the anterograde DiI tracing was used to study the projections from the anteroventral Pe to the GnRH neurons close to the organum vasculosum laminae terminalis (OVLT; Polston and Simerly, 2006). They used CM-DiI (Molecular Probes) to shorten the time of the brain storage in the fixative and for combination of the DiI tracing with GnRH immunocytochemistry. The authors carefully analyzed synaptic oppositions labeled with CM-DiI on the dendrites of GnRH neurons and developed a detailed method for their quantification on the volume reconstructions of the confocal images. This hard work did not reveal representative differences between E19, P0 and P60 brains. Rostral projections of anteroventral Pe to the BSTp and lateral septal nucleus develop later during second and third postnatal weeks. Caudal projections of anteroventral Pe to the parvicellular part of the paraventriculer hypothalamic nucleus were revealed also postnatally. Analysis of these data led authors to the idea that structures containing neurons that express neuroendocrine releasing factors appeared to be innervated first.

## Retrograde tracing of hypothalamic projections to the pituitary and median eminence

The main purpose of our own serial studies was to perform a systematic evaluation of the first steps of the development of main hypothalamic fiber tracts during perinatal ontogenesis in rats using carbocyanine dye tracing. The first studied system was the hypothalamo-pituitary tract that consists of several projection systems well described in the adult. The magnocellular hypothalamic nuclei produce hypothalamic peptidergic hormones (vasopressin and oxytocin) transported by their axons to the posterior pituitary (PL; Hoffman et al., 1986; Hatton, 1990; Falke, 1991). The neurons of the parvicellular hypothalamic nuclei produce adenohypophysiotropic neurohormones, peptides and dopamine and send axons to the median eminence (ME) providing the pathway for the neurohormone transfer via hypophysial portal circulation to the adenohypophysial target cells (anterior lobe, AL; Zimmerman et al., 1984; Swanson, 1986). Some of the parvicellular neurons innervate also the intermediate pituitary lobe (IL).

**Figure 1:**
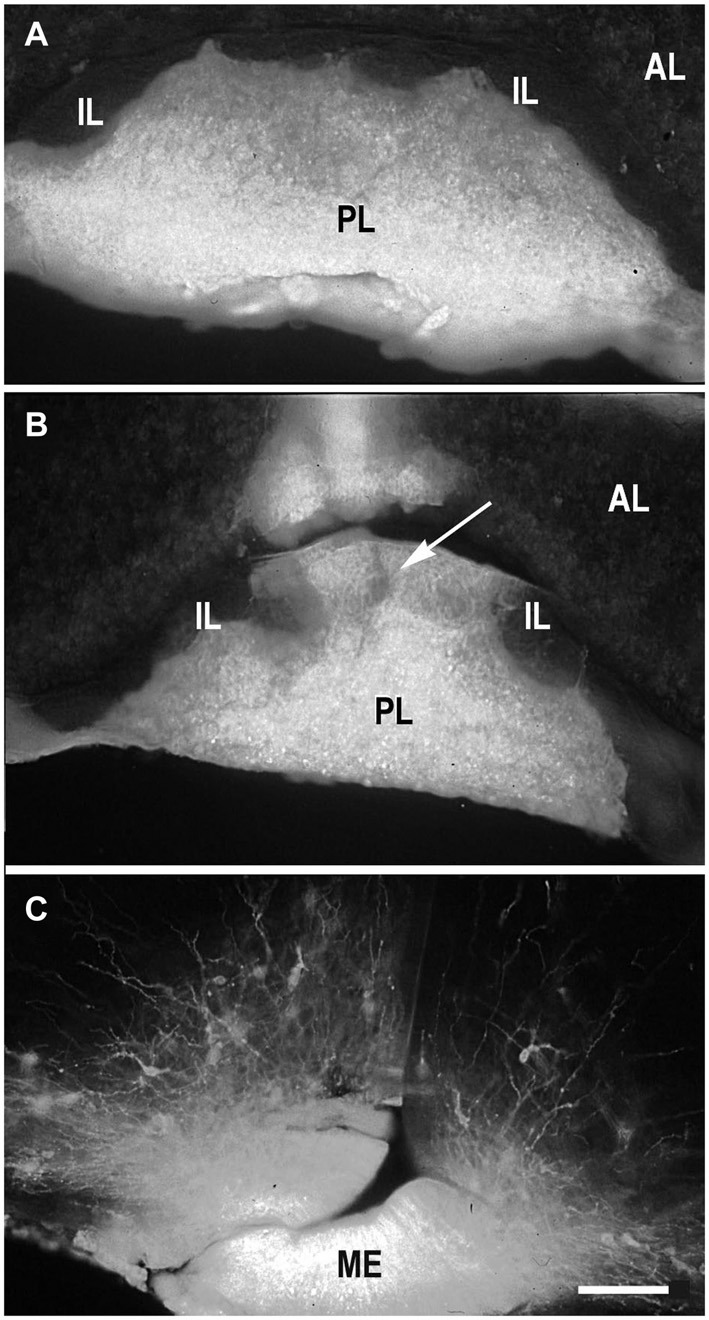
**DiI labeling of the posterior lobe of the pituitary and median eminence**. DiI distribution in the pituitary after insertion into the posterior pituitary lobe (PL) without damage of the boundary between the PL and intermediate (IL) pituitary lobes **(A)**. DiI insertion with labeling in the PL and part (arrow) of IL **(B)**. DiI insertion in the median eminence (ME) with few labeled neurons in the arcuate nucleus **(C)**. AL – anterior pituitary lobe. Scale bar = 100 μm.

Because DiI was used in most of experiments as a retrograde tracer, it was inserted in the terminal regions of the projections of interest (PL, ME or IL) (Makarenko, 2008). DiI was usually distributed strictly in the posterior lobe (Figure 1A) in the cases were the boundary between PL and IL was not damaged (Makarenko et al., 2000). Larger incisions in the PL that penetrated this boundary let the marker spread into both PL and IL (Figure 1B). Separate IL labeling was achieved by pasting DiI crystals on the IL after dissection of the anterior pituitary lobe in postnatal rats (Makarenko et al., 2005; Makarenko, 2008).

### Projections to the posterior pituitary lobe (PL)

**Figure 2:**
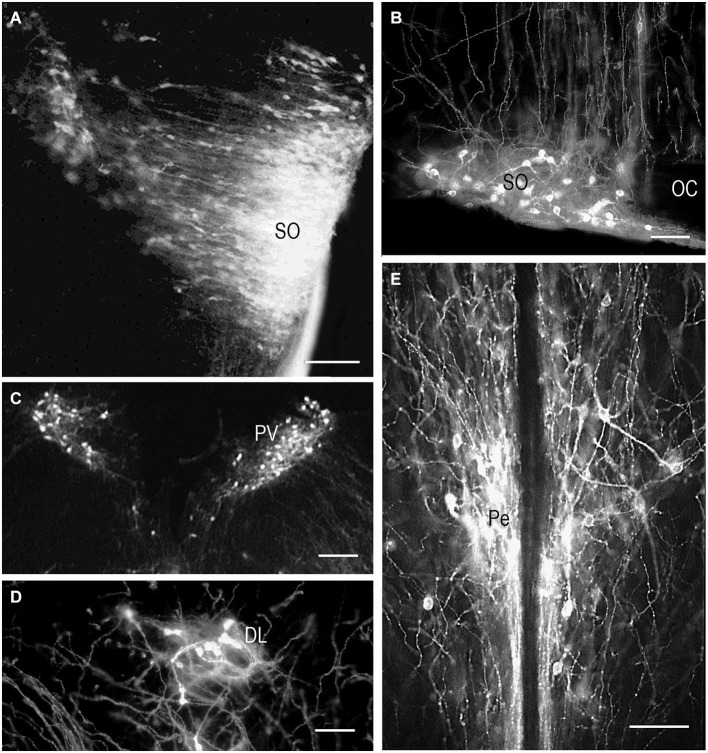
**DiI labeling of the magnocellular hypothalamic neurons in coronal sections**. Low-magnification photomicrographs of the coronal sections of the hypothalamus showing retrograde DiI labeling of the neurons following DiI insertions into the posterior **(A,B,C,D)** and intermediate pituitary lobes on different prenatal stages. **(A)** E15, neurons in the supraoptic nucleus (SO) and wide space of the anterior hypothalamus. **(B)** E20, magnocellular neurons in SO. **(C)** E19, magnocellular neurons in the paraventricular nuclei (PV). **(D)** P9, neurons in the dorsolateral accessory nucleus. **(E)** P10, neurons in the periventricular hypothalamic nucleus after DiI insertion in the intermediate pituitary lobe. Scale bars = 100 μm.

DiI insertion into the PL (Figure 1A) revealed labeled neurons specifically in the regions of presumptive and later differentiated magnocellular peptidergic nuclei on all developmental stages analyzed (Makarenko et al., 2000): supraoptic nucleus (SO) and PV. The most intriguing fact was that on E15–E16 neurons sending axons to the PL were revealed in the anterior hypothalamus from the wall of diamond like swelling of the third ventricle and occupied all space ventrolaterally down to the location of the future SO which looks very large (Figure 2A). This location of the DiI-labeled cells resembles the distribution of magnocellular vasopressin- and oxytocinergic neurons originating from the neuroepithelium of the diamond-like swelling of the third ventricle, in rats at E12–E14 and migrating to the places of their final destination (Altman and Bayer, 1986; Buijs, 1992; Bayer and Altman, 1995). Later on E17–E18, the major DiI-labeled neurons were gathered along the lateral edges of the optic chiasm and tracts, i.e., in the SO (Figure 2B), whereas only a few fluorescent neurons still were seen between the PV. Thus it can be assumed that the labeled cells described earlier between the third ventricle and SO represent migrating magnocellular neurons which axons already reached the posterior lobe of pituitary. Previous immunocytochemical studies failed to visualize vasopressin- or oxytocin- immunopositive axons in the ME and the PL until E17 and E18, respectively (Whitnall et al., 1985; Buijs, 1992).

In contrast to the SO, only occasional labeled neurons were seen in the primordium of the PV at E16 and E17 suggesting that the axons of differentiating neurons of the proper PV reach the posterior lobe significantly later than axons from the SO. These data appear to be consistent with earlier observations of the slower settling and differentiation of magnocellular neurons of the PV when compared to those of the SO (Laurent et al., 1989). On E19 and onwards, the PV contains distinct groups of labeled neurons located in the magnocellular part of the nucleus (Figure 2C). A moderate number of the labeled neurons projecting to PL from the magnocellular part of PV in young rats is in agreement with earlier observations of a rather limited number of axonal projections from the PV to the PL even in adults (Arai et al., 1990).

Magnocellular neurons projecting axons to the PL have been described in adult rats additionally in the accessory peptidergic nuclei (Sofroniew et al., 1980; Ju et al., 1986) and other mammals (Grinevich and Polenov, 1994) were revealed using DiI tracing on the different stages of perinatal development (Makarenko et al., 2002). The retrochiasmatic nucleus that is functionally close to the SO and contains significant number of vasopressinergic neurons sends axons to the PL first on E16–17 and continues development postnatally (Makarenko et al., 2000, 2002). Labeled neurons in the other accessory nuclei such as dorsolateral nucleus (Figure 2D), nucleus of the medial forebrain bundle, circular and commissural nuclei were visualized only postnatally (P2–P10) (Makarenko et al., 2002). Their role is rather questionable and the number of the neurons revealed during perinatal period was significantly lower than in adult rats (Grinevich and Polenov, 1994).

### Hypothalamic projections to the intermediate pituitary (IL)

The IL receives dopaminergic innervation from the periventricular (Pe) and arcuate hypothalamic nuclei in adult mammals (Kawano and Daikoku, 1987; Goudreau et al., 1992; Vanhatalo et al., 1995). As it was very difficult to perform separate DiI labeling of the IL we analized cases with insertions of the marker in the deep incisions through the PL that reached the IL (Figure 1B). As a result labeling in the hypothalamus resembled that described following the DiI insertions in the PL (Makarenko et al., 2000) but with specific visualization of the parvicellular neurons (Makarenko et al., 2005). The first axons arrive to the IL from the Pe in the late prenatal period in coincidence with the first appearance of TH-immunoreactive neurons in the Pe (Jaeger, 1986; Ugrumov et al., 1989). The number of labeled cell bodies in the Pe increased progressively in postnatal rats, in coincidence with the first appearance of the tyrosine hydroxylase-immunoreactive fibers in the IL (Gary and Chronwall, 1992).

Single DiI labeling of the IL in P2–P3 rats revealed neurons in the parvicellular but not magnocellular part of the Pe (Makarenko et al., 2005). Besides, the periventricular and parvicellular PV the labeled neurons were regularly seen in the arcuate nucleus of the neonatal rats following the DiI staining of both IL and PL or only IL. These data shows that IL receives afferents from the parvicellular hypothalamic neurons in rats shortly after the birth. The number of the hypothalamic DiI-labeled neurons and fibers increased progressively from P10 (Figure 2E) to P20. Additional DiI labeled neurons projecting to the IL were revealed in young rats along the dorsolateral border of the ventromedial nucleus. Thus, the hypothalamic parvicellular hypothalamic neurons form innervation of the IL mainly during first three weeks of postnatal life.

### Hypothalamic projections to the median eminence (ME)

Most hypothalamic nuclei projecting to the ME are parvicellular neurons, producing adenohypophysotropic neurohormones, peptides and dopamine (see for reference Ugrumov, 1991). They project axons to the ME providing the pathway for the neurohormone transfer via hypophysial portal circulation to the adenohypophysial target cells (Ugrumov, 1992). The axons containing adenohypophysiotropic neurohormones or the enzymes of their synthesis were first detected by immunocytochemistry not earlier than on E16 (Daikoku et al., 1986; Ishikawa et al., 1986; Okamura et al., 1991). However, immunostaining defined the accumulation of neurohormones in the axons or terminals that could be visualized immunocytochemically but not the first arrival of the axons in the ME. DiI inserted into the median eminence rostrally to pituitary stalk (Figure 1C) labels both fibers and neurons in the hypothalamus from the first rare cells on E14 with subsequent increase during the next few days (Makarenko et al., 2001). They were widely distributed through the hypothalamus and in the ventromedial region of the more rostral forebrain resemble such distribution after PL insertion on E15 because of the DiI labeling of the axons of the hypothalamo-pituitary tract. On E20 most labeled neurons were concentrated mainly in distinct hypothalamic nuclei: the PV (dorsal and medial parvicellular parts), the arcuate nucleus and to a lesser extent in the medial preoptic nucleus, the SO, the diagonal band, and the retrochiasmatic nucleus. In neonates, DiI-labeled neurons appeared additionally in the accessory dorsolateral nucleus, medial preoptic area lateral to the diagonal band, anterior hypothalamic area, and in the anterior Pe. Thus, the axons of differentiating neurons of parvicellular hypothalamic nuclei invade the ME during prenatal development and continue in neonates.

## Retrograde tracing of the lateral septal (LS) projections to the hypothalamus during prenatal development

**Figure 3:**
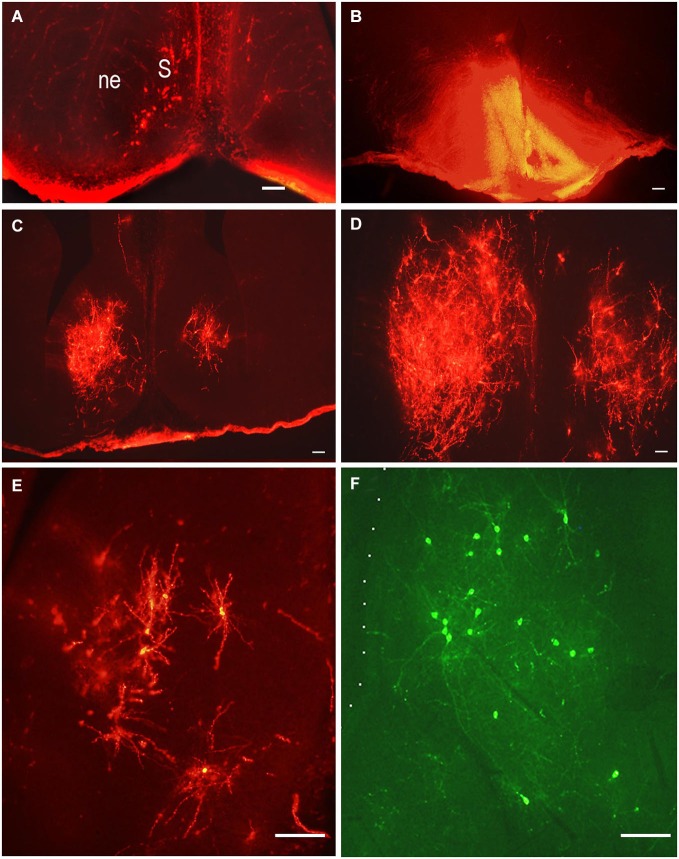
**Lateral septal neurons innervating preoptic area**. Low-magnification photomicrographs of the coronal sections of the septum following carbocyanine dyes insertions in the preoptic area **(B)**. **(A)** E15, neuronal bodies on the left side of the septum after ipsilateral DiI insertion. **(B)** E18, asymmetrical distribution of DiI in the preoptic area; **(C)** and **(D)** E18, distribution of retrogradely labeled neurons in the lateral septal nuclei on the rostral and medial levels. **(E)** E18, DiI-labeled multipolar neurons in the lateral septum at larger magnification. **(F)** E.18, DiA-labeled multipolar neurons in the lateral septum. Scale bars =100 μm.

The septum is an integrative relay station of the limbic system, connecting the limbic forebrain with the hypothalamus and brainstem (Risold and Swanson, 1997; Herman et al., 2002). Its extensive reciprocal connections with the hypothalamus have been visualized and described in adult rats using different methods. Projections from the lateral septal nucleus to the hypothalamus have been described in detail in adult rats (Risold and Swanson, 1997). These connections are topographically organized and belong to specific hypothalamic behavioral systems. The time of origin of the septo-hypothalamic projections has been studied in rats using DiI and DiA as retrograde tracers (Makarenko, 2007). The tracers were applied to different hypothalamic regions: preoptic area, mediobasal hypothalamus and mammillary bodies or adjacent regions of the posterior hypothalamus in rat fetuses on E14.5–E21. The number of retrogradely-labeled neurons revealed in the septum correlates with the region and size of the DiI application (Figures 3B–D). The first septal neurons sending axons to the preoptic area were visualized in the septum on E14.5–E15 but there were no clear differentiation of the septal nuclei and the neuroepithelial layer was very thick (Figure 3A). On E18 the number of the neurons in the lateral septal nucleus projecting to the preoptic area increased significantly and they have surprisingly large dendritic tree when visualized by DiI (Figures 3C–E) that is characteristic for adult rat (Risold and Swanson, 1997). In contrast another carbocyanine dye 4-(4-dihexadecylaminostyryl)-N-methylpyridinium iodide (DiA) visualized using fluoresceine filter revealed the comparable number of neurons in the lateral septum but the marker was accumulated mainly in neuronal bodies with very light staining of the dendrites of septal neurons and afferent hypothalamic axons (Figure 3F). The lateral septum projections to the mediobasal hypothalamus are not so numerous at all studied ages and the number of neurons visualized in the lateral septum was always lower than those projecting to the preoptic area. This was confirmed by a double labeling study, when DiA was inserted into the preoptic region and DiI into the mediobasal hypothalamus on E18–E21 (Makarenko, 2007). These insertions resulted in visualization of few DiI-labeled cells and numerous DiA-labeled neurons in the lateral septal nucleus. Anterogradely labeled fibers were also revealed in the septum on E18 and E20 with concentration in the lateral septal nucleus following DiI insertion in the preoptic area. They can be regarded as afferent hypothalamic fibers innervating the septum. No septal projections to the posterior hypothalamus specifically the mammillary bodies were revealed prenatally, although they were described in adult rats (Risold and Swanson, 1997). According to the data that septal projections to the hypothalamus participate in regulation of several neuroendocrine functions associated with water and salt intake, food intake, thermoregulation, aggressiveness, and sexually related behaviors (for review, see Herman et al., 2002; Sheehan et al., 2004) these results are of interest as providing knowledge that a significant part of septal projections to the preoptic area develop prenatally but other connections are not numerous or even absent before birth.

Additionally our preliminary data on the development of the innervation of the lateral septum by the hypothalamic neurons shows that these axons originate at E18 if not before (Makarenko, 2008) and they are already well differentiated at birth.

## Development of the tracts of the mammillary body (MBO)

In humans, the mammillary bodies are paired brain structures located in the ventral part of the hypothalamus and surrounded by a “capsule” of nervous fibers. They are part of the limbic system and in particular of the classical “Papez circuit” that plays a major role in emotional and motivational activity as well as in memory formation (Mark et al., 1995; Morgane et al., 2005). In rodents, the MBO forms a single, medially located mass of cells sending very specific bilateral efferent projection systems: the short principal mammillary tracts (left and right) divide into two massive efferent fiber bundles, the mamillotegmental tract, directed to the midbrain (Cruce, 1977; Allen and Hopkins, 1990; Hayakawa and Zyo, 1991), and the mamillothalamic tract, innervating the anterior thalamic nuclei (Watanabe and Kawana, 1980; Seki and Zyo, 1984). Afferent projections to the MBO from the ventral and dorsal tegmental nuclei are provided by small fiber system called mammillary peduncle, but not through the mammillotegmental tract (Allen and Hopkins, 1990). In rodents, the MBO is visible on the ventral surface of the hypothalamus and therefore easy to target with DiI crystals. The surrounding capsule prevents diffusion of the marker outside the MBO from E19 on (in the rat). Cases with accidental DiI application in adjacent regions can be used as a control and provide a quite different distribution of labeled structures.

### Mammillotegmental tract (MTeg)

**Figure 4:**
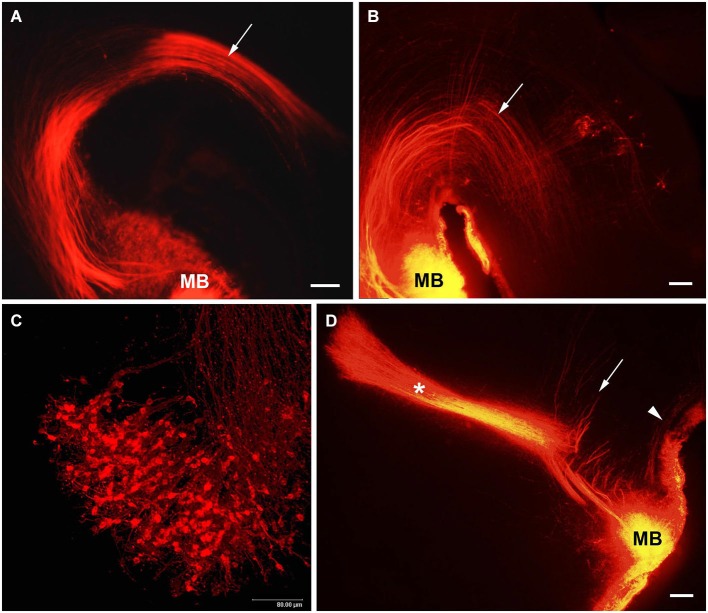
**The MBO axonal tree**. Low-magnification photomicrographs of the sagittal sections of the hypothalamus showing DiI labeling of the fibers of mammillary body tracts following DiI insertion into the mammillary body on different prenatal stages: **(A)** E14, **(B)** E19, **(D)** E21. Mammillotegmental tract–arrow; mammillary peduncle–arrowhead; mammillothalamic tract–asterisk. **(C**) E16, high-magnification confocal image of the sagittal section of the mammillary body showing retrogradely labeled axons and neurons following DiI insertion in the tegmental region. Scale bars: **(A,B,D)** = 100 μm, **(C)** = 80 μm.

**Figure 5:**
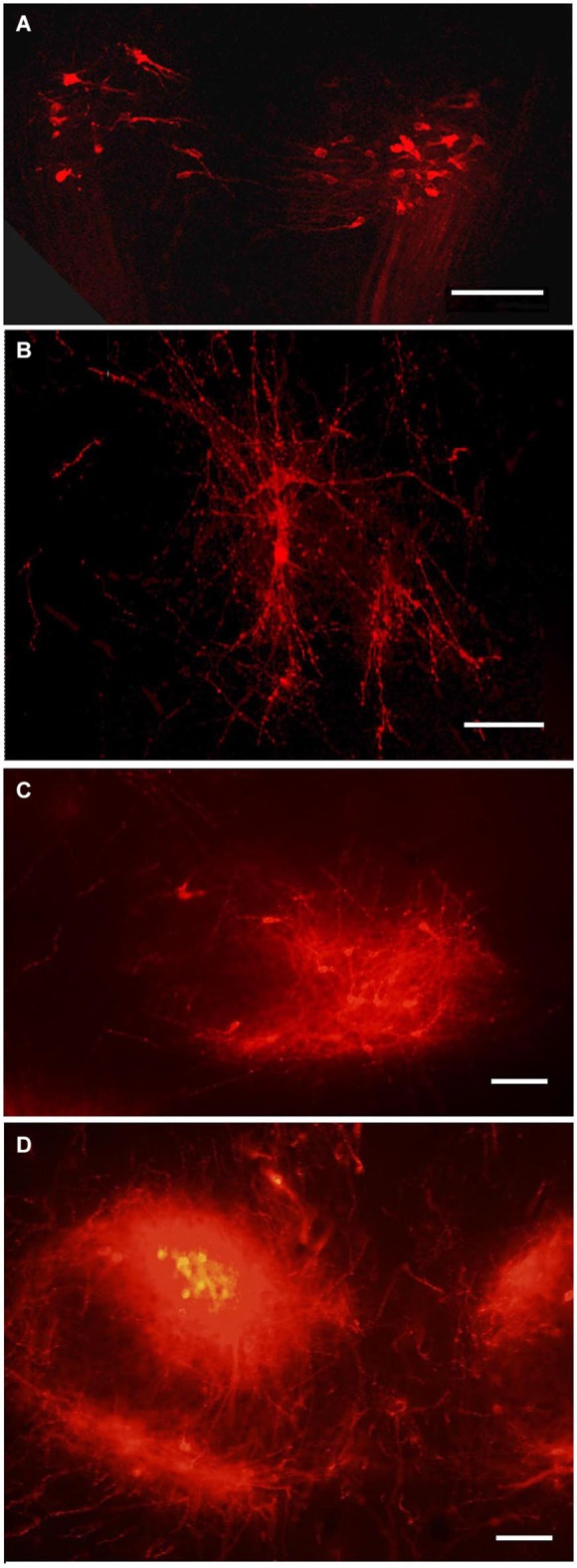
**Tegmental neurons innervating MBO**. DiI labeled neurons in the tegmentum following DiI insertion into the MBO on different prenatal stages: **(A)** E14, bilateral groups of neurons on a coronal section; **(B)** E19, confocal image of the same neurons, in sagittal section; **(C)** P1, the ventral tegmental nucleus on sagittal section; **(D)** P10, ventral tegmental nuclei on coronal section. Scale bars: **(A,B)** = 80 μm, **(C,D)** = 100 μm.

The MTeg is one of the earliest developing brain tracts (Easter et al., 1993; Mastick and Easter, 1996). It consists of MBO efferent axons innervating tegmental nuclei and does not contain reciprocal fibers from the midbrain (Cruce, 1977; Allen and Hopkins, 1990; Hayakawa and Zyo, 1990). We have used anterograde and retrograde DiI tracing to study the MBO-midbrain connections (Alpeeva and Makarenko, 2007). In the rat, at E14 numerous labeled axons of MBO neurons were visible in the principal mammillary tract and in the Mteg, curving along the mesencephalic flexure (Figure 4A). In some cases, a few retrogradely labeled neurons could be seen on coronal brain sections as bilateral groups in the caudal midbrain area (Figure 5A). The labeled growth cones indicate that these are growing efferents. At E19–E21, in cases when the place of DiI crystal insertion was very precisely in the MBO, a distinct group of retrogradely labeled neurons was visualized on sagittal sections in the midbrain, very likely the primordium of the tegmental nuclei (Figure 4B). These neurons have long dendrites and are surrounded by a network of labeled fibers (Figure 5B). DiI application on the same tegmental region visualized retrogradely labeled axons in the MTeg and numerous neuronal bodies in the MBO on E16–19 (Figure 4C) and later.

### Mammillary peduncle (MP)

Afferent axons from the tegmental neurons reach the MBO through the MP (Allen and Hopkins, 1989; Hayakawa and Zyo, 1991). DiI insertions in the MBO labeled both efferents in the MTeg at early prenatal stages and afferents from tegmental nuclei that were visualized from E19 on (Alpeeva and Makarenko, 2007; Figure 4D), although they were not always visible on the same sagittal section. Since retrogradely labeled neurons were observed already at E14–15 among the fibers of Mteg, it is possible that the tegmental afferents develop earlier. Their axons cross the MTeg and formed a bundle (mammillary peduncle) reaching the MBO ventral and lateral to the MTeg. The neurons of the midbrain tegmental area are formed between E13 and E15 in the rat (Altman and Bayer, 1980). At E19, the first fibers from the MTeg turn dorsally and start to form terminal arborizations. Full morphological differentiation of the tegmental nuclei takes place postnatally. From P2 to P10, a single tegmental group divides into two (the dorsal and ventral tegmental nuclei) (Figures 5C,D).

It has been proposed that reciprocal connections between MBO and midbrain tegmental nuclei function as a feedback loop (Gonzalo-Ruiz et al., 1999; Kocsis et al., 2001), which would be an important element of the brain limbic system. DiI anterograde and retrograde tracing has shown that these connections undergo a protracted development from the early emergence of the MTeg through the formation of the mammillary peduncle around E19 to the final differentiation of the tegmental nuclei postnatally.

### Mammillothalamic tract (MTh)

The mammillothalamic tract is a well-organized projection system described in different mammals and innervating three anterior thalamic nuclei (anteromedial, anteroventral and anterodorsal). It is formed by collaterals of the MTeg as has been shown in adult rats with double tracer injections (Hayakawa and Zyo, 1989), and we have confirmed it with DiI in the developing rat (Alpeeva and Makarenko, 2007). The mechanisms and regulation of the development of the MTh has also been investigated by genetic methods in the mouse (Alvarez-Bolado et al., 2000; Szabó et al., 2011).

**Figure 6:**
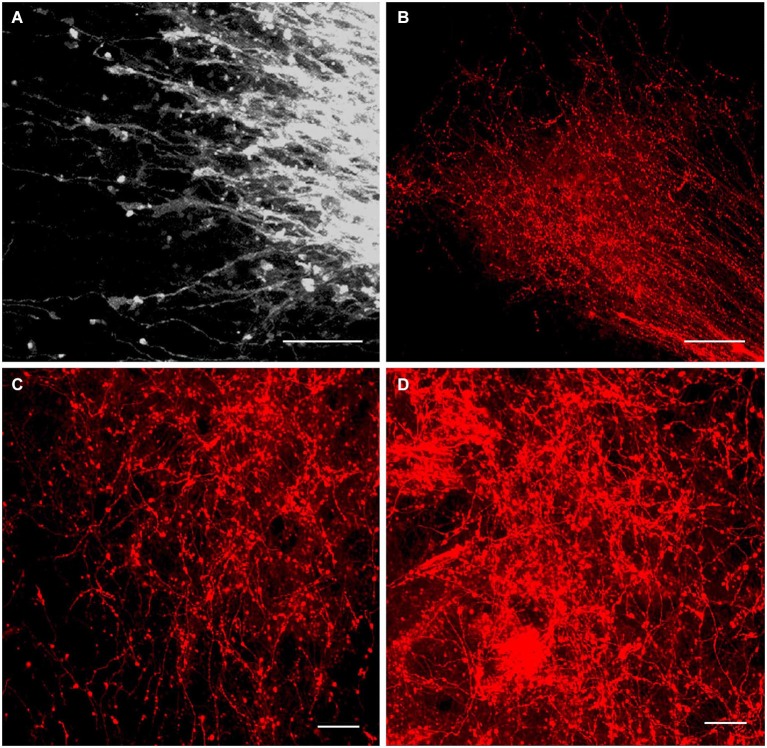
**Innervation of the anterior thalamic complex by the MBO**. High-resolution confocal images of the fine details of the growing mammillothalamic tract and innervation of the anterior thalamic nuclei following DiI insertion in the mammillary body. **(A)** E19.5, axonal growth cones on the top of MTh. **(B)** P2, rostral end of the MTh growing into the anteromedial thalamic nucleus and forms terminal arborizations. **(C)** P4 and **(D)** P10, terminal network of the mammillary body axons in the anteromedial and anteroventral thalamic nuclei. Scale bars: **(A,C,D)** = 20 μm, **(B)** = 80 μm.

The development of the MTh has been studied in the rat using anterograde DiI tracing (Alpeeva and Makarenko, 2009; Makarenko, 2011). From E15 to P5, DiI insertions in the MBO resulted in its distribution in all mammillary body nuclei and projections of the separate MBO nuclei can not be analyzed. The first collaterals of the MTeg became visible at E17 and a short MTh was observed at E18. Numerous fibers grow simultaneously as a tightly packed bundle through the hypothalamus and ventral regions of the thalamus. Confocal scanning of the rostral end of the MTh revealed typical growth cones on the ends of MBO axons (Figure 6A). At E20–21 the MTh grows into the ventromedial region of the anterior thalamic nuclei (Figures 4D, 6B). This fact is in agreement with the evidence that the different nuclear components of the anterior thalamus can be distinguished in the rat at E21 (Coggeshall, 1964). Thus the MBO innervation of the anterior thalamic nuclei develops from late prenatal stages (innervation of the ipsilateral anteromedial nucleus at E20–21). On P1–P2, MBO axons start to innervate the ipsilateral anteroventral thalamic nucleus. At the same time, a separate bundle courses forward in the direction of the anterodorsal thalamic nuclei ipsi- and contralaterally forming the thalamic decussation. At P3–P4, labeled fibers of the MTh filled the whole volume of the anteroventral and began to form the terminal network in the anterodorsal thalamic nuclei. The terminal network consisted of fine beaded nervous processes tightly surrounding all neurons inside the anterior thalamic nuclei (Figure 6C). Immunocytochemical visualization of synapsin, a marker of functioning brain synapses (Melloni and DeGennaro, 1994; Castejón et al., 2004), revealed immunolabeling in the anterior thalamic nuclei as small dots in the neuropil around neurons and density of their distribution correlated with density of the DiI labeled terminal network in each nucleus (Alpeeva and Makarenko, 2009). By P5–P6 and P10 the density of fluorescent Mth terminal arborizations in all three nuclei of the anterior thalamus has grown significantly (Figure 6D).

The differential contribution of MBO subnuclei to the MTh can be distinguished already on P6–P8 (Alpeeva and Makarenko, 2009). Separate labeling of the medial mammillary nucleus or lateral mammillary nucleus with little spread outside revealed that the axons from the lateral MBO innervate both anterodorsal thalamic nuclei (left and right) and the axons from the medial MBO innervated anteroventral and anteromedial thalamic nuclei on the side of DiI insertion. This is in agreement with the data obtained in adult rat (Watanabe and Kawana, 1980; Seki and Zyo, 1984; Hayakawa and Zyo, 1989; Guison et al., 1995; Gonzalo-Ruiz et al., 1998). The adult innervation pattern of the anterior thalamic complex by the MBO is reached around P5–P10.

## Conclusions

The available data, summarized in (Table 1), demonstrate that hypothalamic connections develop with a high degree of spatial and temporal specificity, innervating each target with a unique developmental schedule which in many cases can be correlated with the functional maturity of the projection system.

**Table 1:** **The time schedule of the development of hypothalamic projection systems studied using DiI tracing**.

Projection system	Prenatal (E)							Postnatal (P)											
	Developmental days																		
	E 14	E 15	E 16	E 17	E 18	E 19	E 20	P 0	P 1	P 2	P 3	P 4	P 5	P 6	P 7	P 9	P 10	P 12	P 18
MBO projections to Teg (Mammillotegmental tract)																			
Parvicellular PV, Pe and ARC projections to ME																			
Magnocellular SO and PV projections to PL (Hypothalamopituitary tract)																			
LS projections to PA																			
RC projections to PL																			
MBO projections to AT (Mammillothalamic tract)																			
Teg nuclei projections to MBO (Mammillary peduncle)																			
Accessory hyp. nuclei (DL, MFB, C) projections to PL																			
Parvicellular Pe, Arc and PV projections to IL																			
AVPV projections to GNRH neurons around OVLT																			
SC efferents to main targets																			
Retinal afferents to SC																			
BSTp projections to MPN																			
BSTp projections to AVPV																			
ARC projections to DM, PV																			
ARC projections to LHA, MP																			

It can be useful to distinguish three groups of hypothalamic projection systems according to the period of their formation

1. ***Projections developing prenatally (beginning from E14-E15)***
  - Mammillary body projections to the tegmentum (mammillotegmental tract)
  - Projections of parvicellular hypothalamic neurons (Pe, paraventricular and arcuate nuclei) to the median eminence
  - Projections of magnocellular hypothalamic neurons (SO and PV) to the PL and median eminence
  - Lateral septal projections to the preoptic area
2. ***Projections starting late prenatally and differentiating postnatally***
  - Projections of accessory retrochiasmatic nucleus to the PL
  - Mammillary body projections to the anterior thalamic nuclei (mammillothalamic tract)
  - Projections from the tegmental nuclei to the mammillary body (mammillary peduncle)
  - Projections of the accessory hypothalamic nuclei (dorsolateral, circular, medial forebrain bundle) to the posterior pituitary
  - Projections of the parvicellular hypothalamic neurons (Pe, arcuate and paraventricular nuclei) to the IL
  - Projections of anteroventral Pe to GNRH neurons around OVLT
3. ***Projections developing postnatally***
  - Arcuate nucleus projections to the hypothalamic nuclei (dorsomedial and paraventricular Nuclei and lateral hypothalamic area)
  - Arcuate nucleus projections to the BSTp and lateral septal nucleus
  - Projections of anteroventral Pe to the BSTp, lateral septal nucleus and parvicellular PV
  - Suprachiasmatic nucleus projections to the hypothalamic nuclei, lateral geniculate nuclei, BSTp and lateral septal nucleus
  - GnRH neurons projections to the mediobasal hypothalamus
  - Projections of the BSTp to anteroventral Pe and medial preoptic nucleus
  - Retinal projections to the suprachiasmatic nucleus

## Conflict of interest statement

The author declares that the research was conducted in the absence of any commercial or financial relationships that could be construed as a potential conflict of interest.

## Abbreviations

AL: anterior pituitary lobe
ARC: arcuate hypothalamic nucleus
AT: anterior thalamic nuclei
AVPV: anteroventral periventricular hypothalamic nucleus
BSTp: principal nucleus of the bed nuclei of the stria terminalis
C: circular accessory hypothalamic nucleus
DL: dorsolateral accessory hypothalamic nucleus
DM: dorsomedial hypothalamic nucleus
IL: intermediate pituitary lobe
LHA: lateral hypothalamic area
LS: lateral septal nucleus
ME: median eminence
MFB: medial forebrain bundle accessory hypothalamic nucleus
MBO: mammillary body (bodies)
MPN: medial preoptic nucleus
MTeg: mammillotegmental tract
MTh: mammillothalamic tract
Ne: neuroepithelium
OC: optical commissure
OVLT: organum vasculosum laminae terminalis
PA: preoptic area
Pe: periventricular hypothalamic nucleus
PL: posterior pituitary lobe
PV: paraventricular hypothalamic nucleus
RC: retrochiasmatic hypothalamic nucleus
S: septum
SC: suprahiasmatic hypothalamic nucleus
SO: supraoptic hypothalamic nucleus
Teg: tegmental midbrain nuclei.
